# Overexpression of *KDM6A* in Hepatoma Cells Induces Hepatocytic Differentiation and Attenuates Proliferation Rate, Colony Formation, and Migration Capacities

**DOI:** 10.1155/bmri/5551687

**Published:** 2025-11-15

**Authors:** Mahdieh Hashemi, Mahdi Hesaraki, Roya Ramezankhani, Seyyed Mohammad Yaghoubi, Bahare Shokouhian, Abbas Piryaei, Elham Rismani, Mustapha Najimi, Massoud Vosough

**Affiliations:** ^1^ Department of Regenerative Medicine, Cell Science Research Center, Royan Institute for Stem Cell Biology and Technology, ACECR, Tehran, Iran, royaninstitute.org; ^2^ Department of Molecular Cell Biology-Genetics, Faculty of Basic Sciences and Advanced Technologies in Biology, University of Science and Culture, Tehran, Iran, usc.ac.ir; ^3^ Department of Regeneration Medicine in Wound Healing, Medical Laser Research Center, Yara Institute, ACECR, Tehran, Iran; ^4^ Department of Development and Regeneration, Stem Cell Biology and Embryology, KU Leuven Stem Cell Institute, Leuven, Belgium; ^5^ Immunology Research Center, Tabriz University of Medical Sciences, Tabriz, Iran, tbzmed.ac.ir; ^6^ Department of Biology and Anatomical Sciences, School of Medicine, Shahid Beheshti University of Medical Sciences, Tehran, Iran, sbmu.ac.ir; ^7^ Molecular Medicine Department, Biotechnology Research Center (BRC), Pasteur Institute of Iran, Tehran, Iran, pasteur.ac.ir; ^8^ Laboratory of Pediatric Hepatology and Cell Therapy, Institute of Experimental and Clinical Research (IREC), UCLouvain, Brussels, Belgium; ^9^ Experimental Cancer Medicine, Institution for Laboratory Medicine, Karolinska Institute, Stockholm, Sweden, ki.se

**Keywords:** cancerous phenotype, differentiation therapy, hepatocellular carcinoma, *KDM6A*, lentivirus vector

## Abstract

**Objective:**

Despite the remarkable advances in approved therapeutic approaches, the recurrence rate of hepatocellular carcinoma (HCC) is very high after treatment. Therefore, introducing innovative therapeutic modalities such as targeted molecular therapies is inevitable. Lysine demethylase 6A (*KDM6A*) is a member of the KDM6 family with histone demethylase activity. This gene frequently mutates in different cancers, and its mutations are associated with the increased likelihood of carcinogenesis. This study is aimed at evaluating if inducing *KDM6A* expression could attenuate cancerous features of HCC cells.

**Method:**

A lentiviral‐based vector was used to induce *KDM6A* expression in Huh‐7 cells. The impact of *KDM6A* overexpression on the cancerous phenotype of HCC cells was assessed by measuring proliferation rate, migration and colony formation capacity, and differentiation induction toward hepatocytes.

**Results:**

*KDM6A* overexpression significantly altered cellular morphology, proliferation rate, cell cycle pattern, colony formation, and migration capacity of HCC cells. In addition, induction of differentiation toward hepatocytic fate resulted in down/upregulation of epithelial–mesenchymal transition (EMT) markers associated with the cadherin switch. Furthermore, the expressions of ALB and *HNF4α*, key hepatocytic hallmarks, were increased.

**Conclusion:**

Overexpression of *KDM6A* could be used as a potential noninvasive molecular therapeutic strategy to prevent metastasis and recurrence rate in HCC.

## 1. Introduction

Hepatocellular carcinoma (HCC) is the most common primary liver cancer, which is usually caused by alcohol, hepatotropic viruses, nonalcoholic steatohepatitis, aflatoxin B, and other xenobiotics that lead to inflammation, fibrosis, cirrhosis, and emerging neoplastic cells [1, 2]. HCC is the third leading cause of cancer‐related deaths and the sixth most common cancer worldwide [3]. Early diagnosis and providing effective treatment modalities remain major challenges. Although there are several treatment strategies, the only approved therapy for end‐stage liver diseases is liver transplantation [4]. Despite considerable advances in posttreatment approaches and microscopic surgeries, the possibility of recurrence postsurgery is high due to the molecular heterogeneity, dormant cancer stem cells, efficient angiogenesis in the early stages, and remaining microscopic residues [4–6]. Therefore, researchers are looking for targeted treatments, and the identification of molecular therapeutic targets has received more attention today.

Chromatin remodeling continuously modifies the N‐terminal tail of histones, which is a dynamic and reversible process [7]. In addition, various posttranslational modifications are carried out in the N‐terminal regions of histones containing lysine (K) and arginine (R) residues [8]. One of the important posttranslational modifications on histones is demethylation [9]. *KDM6A* encodes K demethylase 6A, also known as ubiquitously transcribed tetratricopeptide repeat, X chromosome (*UTX*), which catalyzes the removal of trimethyl groups from histone 3 lysine 27 (H3K27) (as illustrated in the graphical abstract) and acts as a tumor suppressor [8]. *KDM6A* plays a substantial role in cell proliferation regulation and division, development, and hematopoiesis [10–12]. It is also reported that *KDM6A* is often mutated which shows different manifestations in different cancer types such as medulloblastoma, colon, non–small cell lung cancer, pancreas, bladder, urinary tract, esophagus, and HCC [13–18].

There have been various studies related to the *KDM6A* and its leading role in carcinogenesis, which have demonstrated considerable results. It plays as a tumor suppressor in HCC, pancreatic cancer, medulloblastoma, and multiple myeloma [2, 16, 19, 20]. In HCC, its downregulation leads to cancer progression via triggering mTORC1 and promoting TGF*β*/SMAD signaling pathways [16, 19]. Conversely, *KDM6A* has an oncogenic role in acute myeloid leukemia and non–small cell lung cancer [17]. The different functions of this gene in the various types of cancers still need to be understood and deciphered. Considering these controversial findings and the potential of *KDM6A* as a therapeutic target, this study is aimed at evaluating whether inducing *KDM6A* expression can attenuate cancerous features of HCC cells. Our findings indicate that *KDM6A* overexpression significantly alters several cancerous characteristics and promotes hepatocytic differentiation, suggesting its potential as a noninvasive molecular therapeutic target to prevent metastasis and recurrence rate in HCC.

## 2. Materials and Methods

### 2.1. Protein–Protein Interaction (PPI) Network Analysis to Evaluate KDM6A in HCC Development

To explore the role of *KDM6A* in HCC, a PPI network was constructed focusing on proteins involved in cell–cell adhesion and epithelial–mesenchymal transition (EMT). Interactions were retrieved from the STRING database, applying a confidence score threshold to ensure data quality. The resulting network was imported into Gephi software (Version 0.9.2) for further analysis. Key topological metrics, including degree centrality (represented by node color), closeness centrality (reflected by node label font size), betweenness centrality (indicated by node size), and eigenvector centrality, were computed to identify hub proteins and network influencers. These parameters enabled the prioritization of candidate genes implicated in *KDM6A*‐mediated HCC development.

### 2.2. Lentiviral Vector Construction

To generate the coding sequence of the *KDM6A* gene, corresponding DNA was amplified out of a human cDNA pool of H9 embryonic stem cells. Subsequently, a second PCR reaction was carried out to add 20 bp overlapping fragments to each end of the final product, with the digested pLenti backbone. The homology sites on the backbone were positioned immediately after the promoter on 5 ^′^ end and inside the P2A sequence on 3 ^′^ end. A DNA fragment with the correct size was then gel‐purified using the PureLink Quick Gel Extraction Kit (Thermo Fisher Scientific). Subsequently, the cloning procedure was performed using 2× Gibson assembly master mix (New England Biolabs) in a final volume of 20 *μ*L, followed by an incubation time of 1 h at 50°C. The final product was transformed into competent *Escherichia coli*. Upon culturing 10 clones for 12 h, the plasmids were extracted using the PureLink HiPure Plasmid Miniprep Kit (Thermo Fisher Scientific) and sent for Sanger sequencing and verification of the correctly assembled vector.

All the PCR reactions were carried out using Q5 Hot Start High‐Fidelity DNA polymerase (New England Biolabs) based on the manufacture’s protocol with a final volume of 50 *μ*L and the following conditions: 98°C for 5 min, 35 cycles at 98°C for 30 s, at temperature annealing for 30 s, and 72°C for 1 kb per minute, and a final 10 min extension at 72°C (Figure S1A, B).

### 2.3. Cell Culture

The Huh‐7 cell line, generated in 1982, originated from a well‐differentiated hepatocyte‐derived carcinoma [21]. It is an immortal cell line composed of epithelial‐like, tumorigenic cells [22, 23]. They are adherent cells and typically grow in monolayer form [24]. Similarly, the HepG2 cell line, established in 1975 from a hepatoblastoma, consists of hepatocyte‐like cells that exhibit some differentiated liver functions. HepG2 cells also grow as adherent monolayers and are widely used as a model for liver metabolism and HCC studies [25, 26].

Human embryonic kidney 293T (HEK293T), HT‐1080, HepG2, and Huh‐7 cell lines were purchased from the Cell Bank of the Royan Institute for Stem Cell Biology and Technology (RI‐SCBT, Iran). The high‐glucose Dulbecco’s Modified Eagle Medium (DMEM) (Gibco, 12800082, United States) was used to culture cells with 1% penicillin/streptomycin (Gibco, 15140122, United States), 1% GlutaMAX (Gibco, 35050038, United States), 1% nonessential amino acid solutions (Gibco, 11140050, United States), 10% FBS (Gibco, 10270106, United States) for HepG2 and Huh‐7 cells, and 15% FBS for HEK293T and HT‐1080 cells and incubated at 37°C with 5% CO_2_. In all experiments, 10–15 days after antibiotic‐based selection, cells were used for molecular and functional assessments (Figure S1C).

### 2.4. Lentivirus Generation

The pLenti‐*KDM6A* vector was used to make a *KDM6A*‐expressing vector by constructing and inserting a *KDM6A*‐encoding sequence. For the purpose of lentivirus generation, HEK293T cells were cultured in 10 cm plates 1 day before transfection and transfected at 70% confluency using 12 *μ*g packaging vectors (psPAX2 and pMD2.G), 6 *μ*g pLenti‐*KDM6A*, 30 *μ*L lipofectamine, and 30 *μ*L P3000 (Thermo Fisher, 3000150, United States) in 1200 *μ*L serum‐free high‐glucose DMEM. The plasmid mix was incubated at RT (room temperature) for 30 min and added dropwise to the cells. One day (24 h) posttransfection, the medium was renewed. The lentiviral supernatant medium was harvested 48 h after transfection using 0.45 *μ*m cellulose acetate membrane filters (FilterBio, FBS25PES045S, China) and stored at −80°C. HT‐1080 cells (data not shown) were used to evaluate virus titer according to the Invitrogen company protocol (Catalog No. A11141).

### 2.5. Transduction

Huh‐7 cells were seeded to 15 × 10^4^ density on 6 cm plates. The cells were transduced when reaching 50%–60% confluency, with viral supernatants and multiplicity of infection (MOI):3 in the presence of polybrene (4 *μ*g/mL) and were incubated for an overnight at 37°C. Two days from the transduction, cells were selected with puromycin (Sigma, P8833‐25MG, United States) (10 *μ*g/mL) treatment for 4–10 days. Figure 1a represents the protocol or timeline of this study. Huh‐7 cells were transduced using the lentiviral vector *KDM6A* (Figure 1b) on Day 2.

Figure 1The physiologic function of the KDM6A and its correlation with other proteins. (a) Timeline of this study. (b) Schematic illustration of *KDM6A* vector containing puromycin resistance gene. (c) Protein–protein interaction network of key hepatocyte differentiation and EMT‐related proteins. Figure designed by Gephi software.

**Figure:**
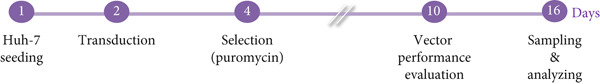
(a)

**Figure:**
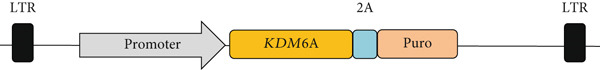
(b)

**Figure:**
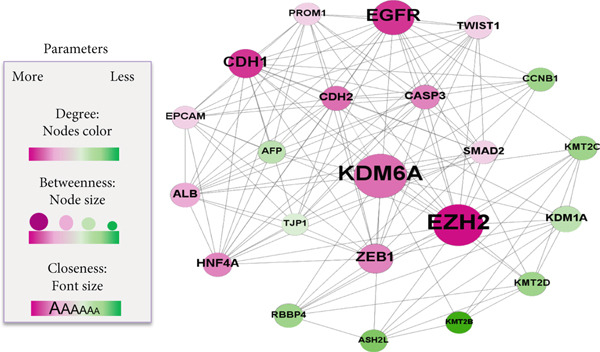
(c)

After constructing the lentiviral vector, we performed the necessary checks to ensure that the empty vector had nonsignificant differences with the control group (CTRL). It should be mentioned that gene expression, proliferation, and colony formation capacity assays were all investigated and nonsignificant differences were noticed in the empty vector group with the CTRL group (Figures S1D, S1E, and S1F).

### 2.6. Bromodeoxyuridine (BrdU) Assay

Huh‐7 cells were plated at 16 × 10^3^ cells/cm^2^ in 4‐well plates. After 24 h, according to the manufacturer’s protocol (Roche, Sigma‐Aldrich, 11296736001, United States), medium containing 5‐BrdU solution was added, and cells were incubated for 60 min at 37°C and fixed with ethanol fixative (composed of glycine solution and absolute ethanol, pH 2) for 20 min at −20°C. Then, cells were treated with anti‐BrdU solution and incubated for 30 min at 37°C. Finally, the cells were treated with antimouse fluorescein antibody and incubated for 30 min. Hoechst dye was used for 30 min to counterstain the nuclei. Fluorescence photos were taken using an IX71 microscope (Olympus, IX7, Japan). ImageJ software was used to quantify the results based on the number of nuclei (blue) and the number of cells that have reached the proliferative phase and are positive for BrdU (green).

### 2.7. MTS Assay

Transduced Huh‐7 cells were seeded at 15 × 10^3^ cells/cm^2^ in a 96‐well plate. After 24 h, medium containing MTS reagent (Promega, G1111, United States) (5 mg/mL) at a ratio of 1:5 was added. Cells were incubated for 2 h at 37°C. Thereafter, absorbance was measured using (Thermo Fisher Scientific, SN:1500‐905, Finland) plate reader at 490 nm.

### 2.8. Colony Formation Assay

One hundred and ten cells per square centimeter of transduced Huh‐7 cells were cultured in a 6‐well plate, and the colony formation was analyzed according to Franken et al. [27]. After 12 days, the emerged colonies were fixed using methanol. The colonies were stained with 1% crystal violet solution. ImageJ software was used to quantify the results based on the number of colonies and their respective area.

### 2.9. Wound Healing Assay

Transduced Huh‐7 cells were cultured at a density of 5 × 10^4^ cells/cm^2^ in a 12‐well plate for 1 day. To eliminate the effect of proliferation on the migration rate, the cells were treated with 1% mitomycin C (1 mg/mL) (Sigma, Cat. No. M0503, United States) for 3 h. Then, a straight scratch was made using a yellow (100 *μ*L) micropipette tip. In the scratch assay, photographs were captured at 0, 24, and 48 h using an inverted light microscope (Olympus, CKX41, Japan). Using ImageJ software, the area of the scratch was measured and quantified.

### 2.10. RNA Extraction and RT‐qPCR

A total number of 11 × 10^4^ Huh‐7 cells/cm^2^ were transduced and cultured in a 6‐well plate for 1 day. Total RNA was extracted using TRIzol reagent (KIAzol, 011013) in accordance with the manufacturer’s protocol. Similarly, total RNA was extracted from normal liver, HepG2, and Huh‐7 cell lines to evaluate *KDM6A* gene expression. Two microgram cDNA was synthesized using the cDNA synthesis kit (Parstous, A101161, Iran) based on the manufacturer’s protocol and diluted 1:10 before being subjected to qPCR. The generated cDNA was mixed with Power SYBR Green Master Mix (Ampliqon, Cat. No. A325402, Denmark) and target‐specific primers in triplicate. Transcript levels were normalized to the *GAPDH* mRNA expression levels and calculated using the 2^−(*Δ*CT)^ method. The qPCR was performed using the StepOnePlus Real‐Time PCR system (Applied Biosystems 7500, SN: 3831122570, Singapore). The primers used in this study are listed in Table S1.

### 2.11. ELISA

A total number of 28 × 10^3^ Huh‐7 cells/cm^2^ were transduced and cultured in 12‐well plates and incubated to attach and proliferate for 5 days. The supernatants of the cells were collected and assessed for the albumin (ALB) and alpha‐fetoprotein (AFP) concentrations using the ALB‐ELISA (Invitrogen, EHALB, Canada) and AFP‐ELISA (Pishtazteb, 01003, Iran) kits according to the manufacturer’s instructions. Data were normalized to total protein (nanograms per milliliter).

### 2.12. Immunofluorescence

The density of 43 × 10^3^ cells/cm^2^ transduced Huh‐7 cells were cultured in 4‐well plates for 1 day. The cells were fixed with 4% paraformaldehyde, washed by PBS‐Tween (0.05%), and treated by Triton (0.1%); then, FC receptor was blocked by host serum and finally washed by PBS‐Tween (0.05%) and incubated with the primary antibodies for an overnight at 4°C zonula occludens protein 1 (ZO1) (1:100; ab216880; Abcam, United States) and hepatocyte nuclear factor 4 alpha (HNF4*α*) (1:200; Abcam, ab41898; United States). The cells were washed with PBS‐Tween (0.05%) two times and incubated with Goat antimouse IgG (DyLight 488) (for anti‐HNF4*α*, Abcam, ab96879, United States) and Goat antirabbit IgG (DyLight 594) (for anti‐ZO1, Abcam, ab98509, United States) secondary antibodies (1:700), respectively, for 2 h at 37°C. After a washing step with PBS‐Tween (0.05%), DAPI was incubated for nuclear counterstaining. Finally, the stained samples were visualized with a fluorescent microscope (Olympus, IX71, Japan). ImageJ software was used to quantify the results.

### 2.13. Western Blotting

A total number of 11 × 10^4^ Huh‐7 cells/cm^2^ were transduced and cultured in a 6‐well plate for 1 day. Total proteins were extracted using RIPA lysis buffer supplemented with phenylmethylsulfonyl fluoride. Total cell lysates were centrifuged at 10,000 × g for 15 min at 4°C, and the supernatants were collected. The protein concentrations of the supernatants were determined using the BCA. Proteins (20 *μ*g per lane) were separated via 12% SDS‐PAGE and transferred onto nitrocellulose membranes. The membranes were blocked with 1% dry milk at 25°C for 60 min. Subsequently, the membranes were incubated at 4°C overnight with anti‐HNF4*α* (1:1000; ab41898; Abcam, United States) and anti‐*β* actin (1:1000; Proteintech, 60008‐1‐Ig, United States) primary antibodies. Secondary antibody goat antimouse IgG FC (1:50,000; Invitrogen, 31437, United States) was incubated at 25°C for 60 min. ImageJ software was used to quantify the bands.

### 2.14. Statistical Analysis

All experiments have been conducted with three biological and at least two technical replicates. The quantitative data were analyzed using *t*‐test to compare two groups or one‐way ANOVA for the comparison of more than two groups and two‐way ANOVA for the wound healing assay using Prism 8 software. The presentation of data was formatted as mean ± standard deviation (SD), and significant differences of the results were categorized at the *p* < 0.05 level. The graphs were drawn by Prism 8 software.

## 3. Results

### 3.1. PPI Network Analysis Identifies KDM6A’s Potential Role in EMT and Cell–Cell Connection in HCC

PPI network of the main proteins that is involved in the process of EMT and cell–cell connection indicated that the main proteins play a crucial role in HCC progression. The analysis highlighted three important proteins: EZH2, KDM6A, and EGFR. Due to the few published research articles and the controversial and ambiguous data regarding the role of KDM6A in the development of HCC, we decided to evaluate its gene and function in the HCC cell model. For the purpose of investigating the associations and finding the hub proteins involved in the differentiation and EMT process, to build a PPI network, the STRING database was used (Figure 1c). The network was drawn by Gephi, and “centrality parameters,” including degree (indicated by the node’s color), betweenness (indicated by the node’s size), closeness (indicated by the font’s size), and eigenvector, were calculated with Gephi software (Figure 1c). The degree of KDM6A was 29 and ranked fourth in the network. KDM6A, exhibiting the highest betweenness value of 50.99, ranked fourth in closeness with a score of 0.77, and possessed an eigenvector parameter of 0.67, was identified as a hub protein within the analyzed network.

### 3.2. *KDM6A* Expression in Cell Lines

Prior to transduction, the expression levels of KDM6A were assessed across three groups of normal primary hepatocytes, Huh‐7, and HepG2 cell lines to identify the most appropriate model for subsequent experiments. Gene expression analysis revealed a significant downregulation of KDM6A in both HepG2 and Huh‐7 cell lines compared to normal liver tissue. Based on these findings, Huh‐7 cells were selected as the most suitable cell line to address the study hypothesis (Figure S1G,H). A temporal analysis at 48 and 72 h and 10 days in Huh‐7 indicated a significant increase in *KDM6A* and Cadherin 1 (*CDH1*) expression, elucidating the dynamics of phenotypic changes (Figure S1I).

### 3.3. Efficient KDM6A Transduction Leads to Significant Upregulation in Huh‐7 Cells

To confirm the transduction efficiency of the *KDM6A* vector in Huh‐7 cells, real‐time quantitative PCR was performed. The expression of *KDM6A* was quantified and normalized to two housekeeping genes, *GAPDH* and *HISTON H1b1*. *KDM6A* expression was evaluated in Huh‐7 cells transduced with the lentiviral vector, compared to an untreated CTRL group and normal liver samples. The results showed a significant reduction of *KDM6A* expression in the CTRL group, whereas the transduced cells exhibited robust upregulation of *KDM6A* compared to the normal liver sample. The gene expression results confirmed an upregulation significantly in the *KDM6A* expression in the transduced Huh‐7cells compared to the CTRL one (Figure 2a).

Figure 2The effects of *KDM6A* overexpression on transcription profile of cancerous cells. (a) Using qRT‐PCR, we evaluated the efficiency of transduction. The expression of *KDM6A* was evaluated and normalized to *GAPDH* and *HISTON H1b1* housekeeping genes. (b) The *KDM6A* overexpression‐related regulation of the mRNA expression levels of proliferation (*CCNB1*) and stemness (*PROM1*) markers demonstrated with reduce expression of CCNB1 and PROM1 in Huh‐7 cells compared to the CTRL group. (c) The effect of *KDM6A* overexpression on epithelial (*CDH1*) and mesenchymal (*CDH2*) genes involved in the adherens junctions (*ZO1*), migration (*ZEB2*), EMT process, and switch cadherin in Huh‐7 cell lines demonstrated with upregulated of *ZO1* and *CDH1* and downregulation of *ZEB2* and *CDH2*. (d) The effect of *KDM6A* overexpression on expression levels of hepatic markers demonstrated an upregulation of *HNF4A* and *ALB* in Huh‐7 cells compared to the CTRL group. *GAPDH* was used as housekeeping genes and data normalized to the CTRL group. Data are presented as the *t*‐test analysis, the mean ± SD for three biologically independent repeated experiments.  ^∗∗∗∗^ *p* value < 0.0001;  ^∗∗∗^ *p* value < 0.001;  ^∗∗^ *p* value < 0.01;  ^∗^ *p* value < 0.05.

**Figure:**
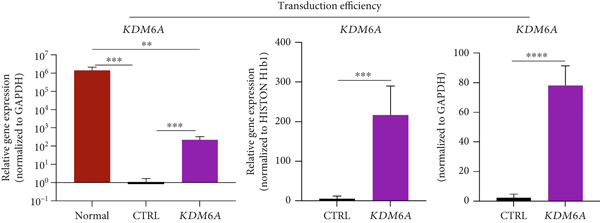
(a)

**Figure:**
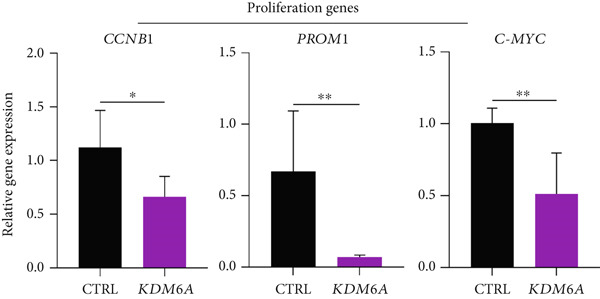
(b)

**Figure:**
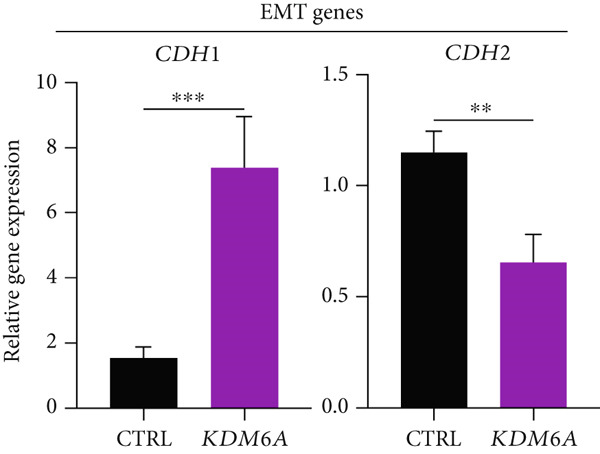
(c)

**Figure:**
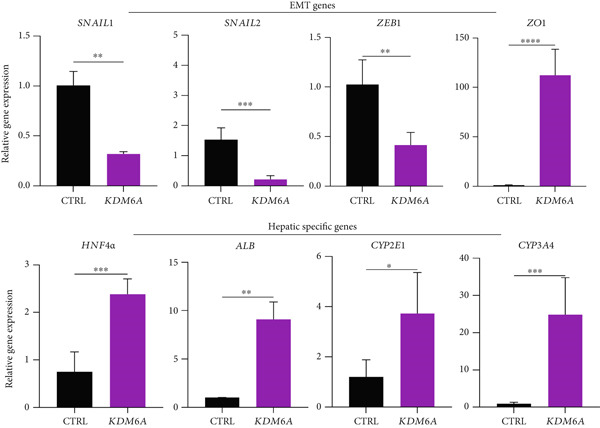
(d)

### 3.4. *KDM6A* Overexpression Inhibits Proliferation and EMT Markers and Induces *HNF4α* and *ALB* Expression

To evaluate the regulatory role of the KDM6A’s gain of function impact on the genes mainly involved in cancerous phenotypes, the transcripts of cyclin B1 (*CCNB1*), Prominin 1 (*PROM1*), *c-MYC*, *CDH1*, Cadherin 2 (*CDH2*), zinc‐finger E‐box‐binding homeobox 1 (*ZEB1*), *SNAIL1*, *SNAIL2*, *ZO1*, *HNF4α*, and *ALB* in *KDM6A*‐overexpressing group were assessed. The mRNA expression levels of *PROM1* (a stemness marker), *CCNB1* (a cell cycle regulator), and c‐MYC (a marker of proliferation and EMT) were significantly downregulated in *KDM6A*‐overexpressing Huh‐7 cells compared to the CTRL group (Figure 2b).

One of the hallmarks of the EMT–mesenchymal–epithelial transition (MET) process is to study the “cadherin switch.” The transcripts of *CDH1* and *CDH2* were evaluated. The gene expression results demonstrated that following *KDM6A* overexpression, the *CDH1* transcripts were significantly upregulated, while those of *CDH2* were dramatically downregulated in the *KDM6A*‐overexpressing cells compared to the CTRL group. Furthermore, the expression of *SNAIL1*, *SNAIL2*, and *ZEB1*, key contributors to the EMT process, was significantly decreased, while the expression of *ZO-1*, a junctional complex protein, was significantly increased in *KDM6A*‐overexpressing cells compared to the CTRL group (Figure 2c). Regarding hepatocyte differentiation markers, the *KDM6A*‐overexpressing group showed significantly higher levels of ALB and *HNF4α*, as well as increased expression of *CYP3A4* and *CYP2E1*, indicating enhanced metabolic function and cell maturation compared to the CTRL group (Figure 2d).

### 3.5. *KDM6A* Overexpression Induced the Protein Expression of ZO1 and HNF4*α*

The protein expressions of ZO1 and HNF4*α* were assessed using immunofluorescence. Quantifying the immunopositive cells indicated that there are significantly higher percentages of ZO1 and HNF4*α* positive cells in the *KDM6A* overexpressed group in comparison to the CTRL group (Figure 3a,b). As a result, it can be stated that the *KDM6A* overexpression induces the expression of ZO1, which may be effective in the EMT process and proliferation by affecting the process of tight junctions and cell–cell junctions and also reducing the migration of cancer cells. To further validate mRNA expression, we evaluated HNF4*α* protein expression as the main transcription factor of hepatocyte differentiation, and a Western blot was used. Quantification of the protein levels of this transcription factor demonstrated a significantly higher level in the *KDM6A* overexpressed group as compared to the CTRL group (Figure 3c).

Figure 3Overexpression of *KDM6A*‐induced hepatocyte differentiation and functionality. (a) Immunofluorescence staining indicated a significant increase in ZO1 protein that is a structural junctional protein, functional hepatocyte polarity, and cell–cell adhesion and (b) increase in HNF4*α* protein that critical role in the maturation and functionality of hepatocytes after *KDM6A* overexpression of Huh‐7 cells compared to the CTRL group. (c) Western blot analysis indicated that transduction of Huh‐7 cells by plenti‐KDM6A vector led to HNF4*α* of upregulated (normalized to ACTB) and quantified using ImageJ software. (d) ELISA analysis indicated that *KDM6A* overexpression caused escalated albumin and decreased AFP secretion compared with the CTRL group in the Huh‐7 cells. Data are presented as the *t*‐test analysis, the mean ± SD for three biologically independent repeated experiments.  ^∗∗∗∗^ *p* value < 0.0001;  ^∗∗∗^ *p* value < 0.001;  ^∗∗^ *p* value < 0.01;  ^∗^ *p* value < 0.05.

**Figure:**
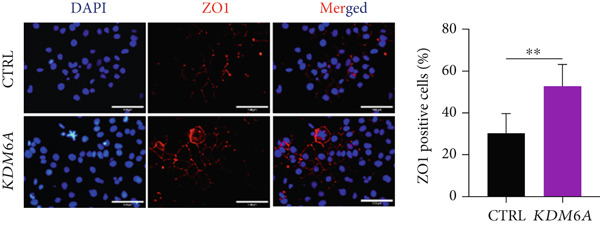
(a)

**Figure:**
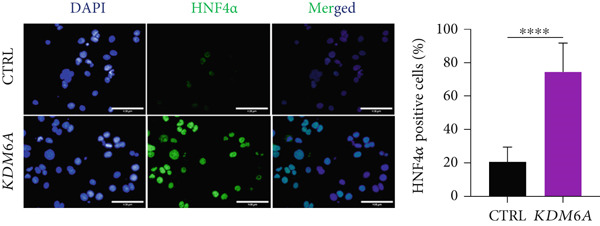
(b)

**Figure:**
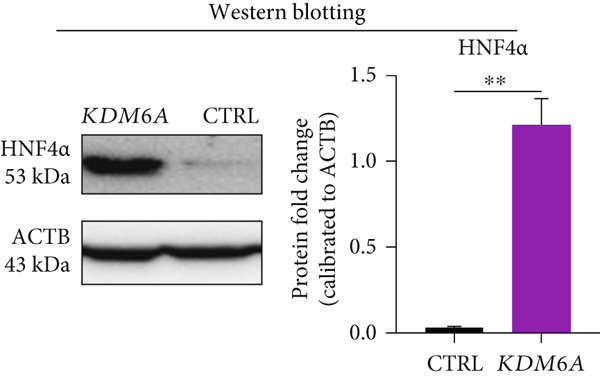
(c)

**Figure:**
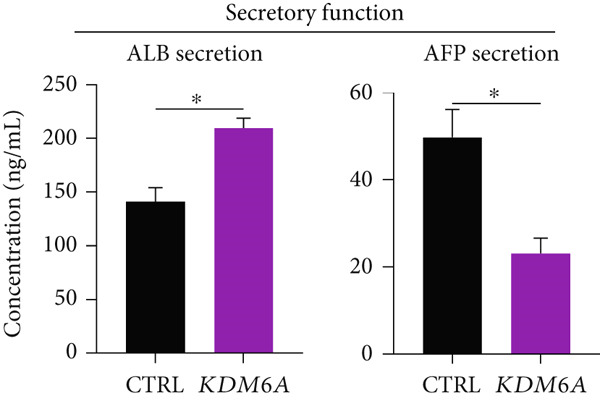
(d)

### 3.6. *KDM6A* Overexpression Increased the Secretion of ALB While Decreasing of AFP

To evaluate the impact of *KDM6A* overexpression on Huh‐7 functionality, the amounts of ALB and AFP secretion were evaluated using an ELISA. The results showed a considerable increase in the secretion of ALB concomitantly with a significant decrease in AFP secretion following overexpression of the *KDM6A* compared to the CTRL group (Figure 3d).

### 3.7. *KDM6A* Overexpression Inhibits Proliferation and Colony Formation Capacity in Hepatoma Cell Line

The growth and cell proliferation rates were assessed using the BrdU assay; we indicated that *KDM6A* overexpression markedly inhibited proliferation compared to the CTRL group (Figure 4a). Moreover, colony formation assays indicated that *KDM6A* upregulation resulted in a dramatic decline in cell proliferation and colony‐forming ability when compared to the CTRL group (Figure 4b). In this regard, some proliferation parameters, in terms of colonies’ areas and plating efficiency percentage and surface area of colonies, were significantly reduced in the *KDM6A*‐overexpressing group compared to the CTRL group (Figure 4c,d). Similarly, the MTS assay indicated that the proliferation capacity of *KDM6A*‐overexpressing was significantly lower than the CTRL group (Figure 4e).

Figure 4Functional analysis of the *KDM6A* overexpression resulted in impaired proliferation rate, colony formation capacity, viability, and migration ability of the HCC cell line. (a) BrdU assay measured the proliferation rate, and we observed a decrease in the cell proliferation after *KDM6A* overexpression of Huh‐7 cells compared to the CTRL group. (b) The colony formation assay showed reduction in the colony formation of the cells after *KDM6A* overexpression. (c) The average area of colonies and (d) plating efficiency percentage was decreased in *KDM6A* overexpression group compared to the CTRL group. (e) MTS assays: decreased the proliferative ability compared to the CTRL group. (f) The wound healing assay showed a reduction in the migration and area percentage (g) after *KDM6A* overexpression compared to the CTRL group. Data are presented as mean ± SD for three biologically independent repeated experiments.  ^∗∗∗∗^ *p* value < 0.0001;  ^∗∗∗^ *p* value < 0.001;  ^∗∗^ *p* value < 0.01.

**Figure:**
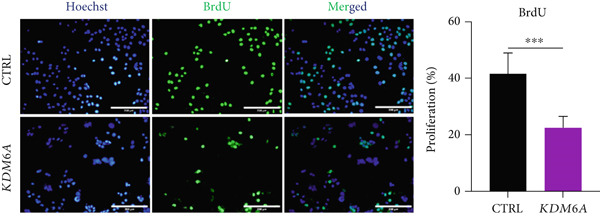
(a)

**Figure:**
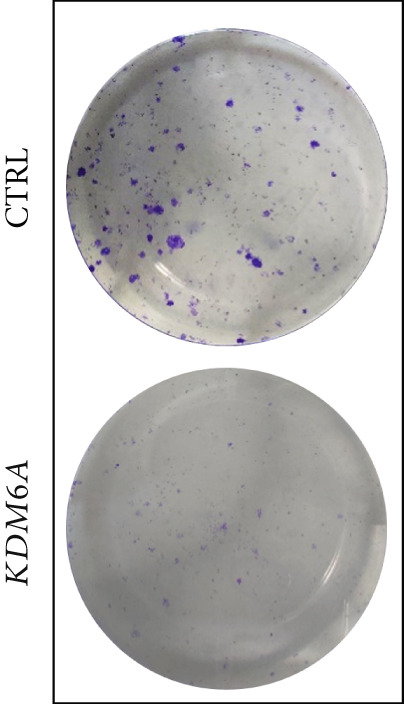
(b)

**Figure:**
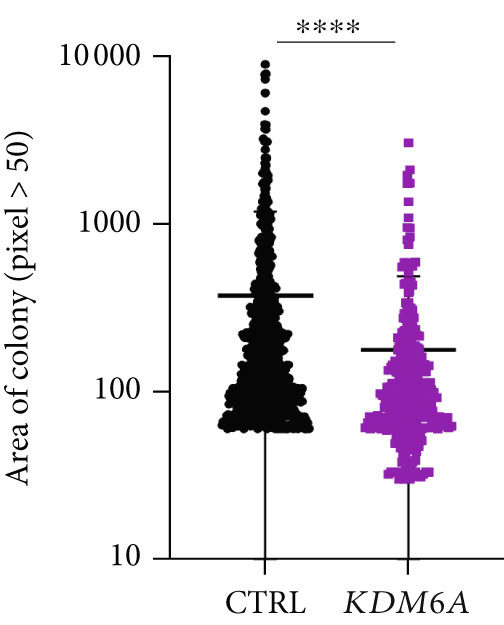
(c)

**Figure:**
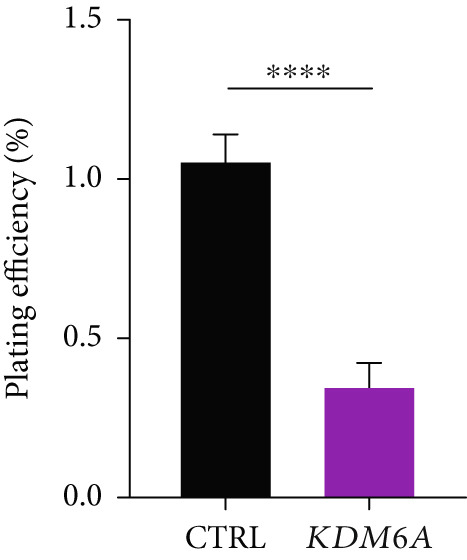
(d)

**Figure:**
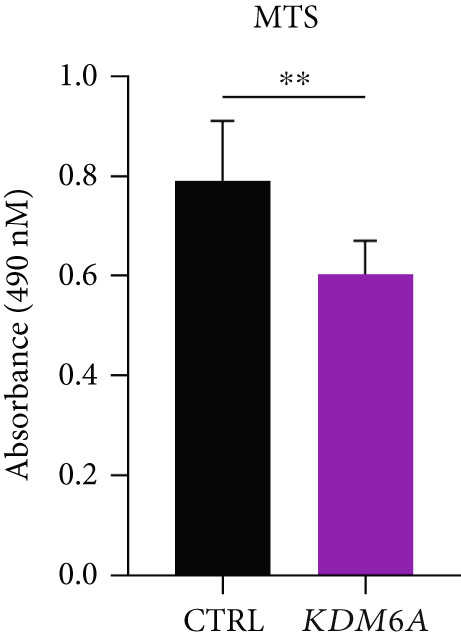
(e)

**Figure:**
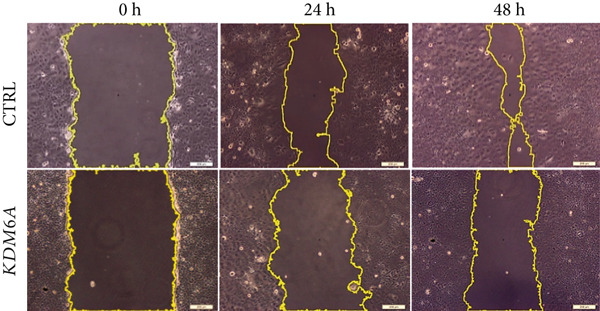
(f)

**Figure:**
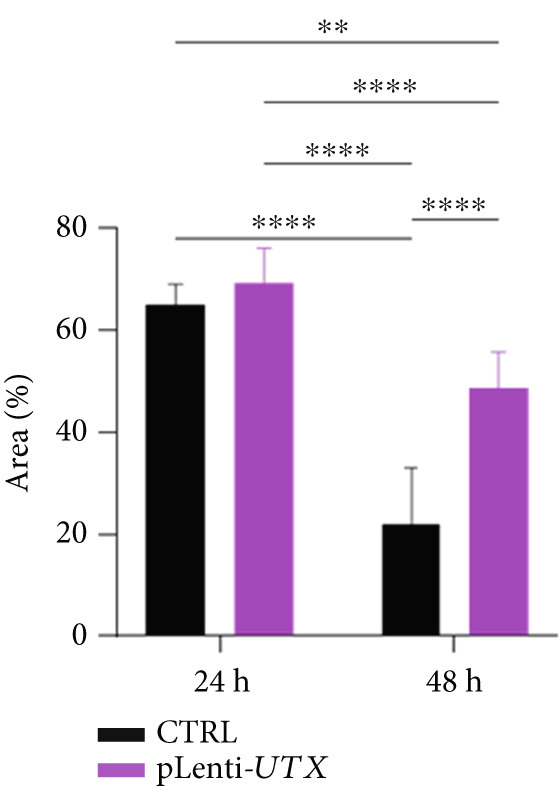
(g)

### 3.8. *KDM6A* Overexpression Inhibits HCC Cell Migration

We used the wound healing assay to assess cell migration and inhibited cell proliferation using 1% mitomycin C. The results showed that the scratch area percentage of the *KDM6A*‐overexpressing Huh‐7 cells was significantly lower than the CTRL group (Figure 4f,g). Also, our results of a follow‐up to investigate the morphological changes using an invert microscope showed that in the *KDM6A*‐overexpressing group, the cells showed round polygonal shapes, the nucleus and nucleoli became smaller, and the cells’ cytoplasm was higher compared to the CTRL cells (Figure S2).

## 4. Discussion

Despite the recent advancements in the treatment of HCC, adverse effects, high recurrence rate, resistance to treatment, and diversity in the therapeutic responses have limited its broad clinical applications. There remains an urgent need for innovative, targeted molecular therapies to improve outcomes by specifically inhibiting invasion, migration, and proliferation markers in HCC patients [4, 5, 28–30]. In the current research, we investigated *KDM6A* overexpression on the cancerous phenotype of transduced Huh‐7 cells.

KDM6A functions as a histone H3K27me3 demethylase and a chromatin remodeler with broad transcriptional influence affecting development, proliferation and development, hematopoiesis, and differentiation [10, 12, 31–34]. Importantly, KDM6A exhibits a dual role in cancer biology. It acts as a tumor suppressor in several cancers including liver, medulloblastoma, and bladder cancers, where its expression is frequently downregulated or mutated [16, 19, 35, 36]. Conversely, it displays oncogenic activity in breast, colorectal, and lung cancers with elevated expression [14, 17, 18, 37]. In HCC, the literature presents conflicting evidence: Some studies report KDM6A downregulation correlated with enhanced tumorigenesis and treatment resistance, while others describe increased expression in tumor tissues [38, 39]. This complexity underscores the need for further understanding of KDM6A’s context‐dependent function in liver cancer.

Our PPI network analysis indicated that KDM6A’s high degree of connectivity within the network, particularly its interactions with proteins involved in differentiation and EMT, thereby supports its involvement in HCC progression. Given that KDM6A demethylates H3K27me3 (trimethylation of K 27 on histone H3), counteracting EZH2/PRC2’s methyltransferase activity, it serves as a key epigenetic regulator influencing chromatin plasticity and gene expression programs [13, 40–42]. Indeed, modulation of chromatin erasers like *KDM6A* can reprogram entire gene networks, offering genome‐wide influence and mechanistic clarity that surpasses factors like *EGFR* [34]. Despite extensive studies on *EGFR* and *EZH2*, *KDM6A* remains less understood and holds greater potential for novel insights, warranting comprehensive investigation.

Experimentally, both Huh‐7 and HepG2 cells showed reduced baseline KDM6A expression compared to normal liver, with Huh‐7 chosen for overexpression studies due to its lower basal level, facilitating detectable effects. Furthermore, lentiviral vectors were utilized to ensure *KDM6A* stable expression and a higher transduction rate, particularly in hard‐to‐transfect cells such as HepG2 cells.

In our study, *KDM6A* overexpression led to significant impairment of HCC cell proliferation rate, colony formation, and growth, reflected by reduced expression of stemness and proliferation markers *PROM1*, *c-MYC*, and *CCNB1*. The downregulation of *c*‐*MYC*, a major regulator of cell cycle and proliferation that promotes *CCNB1*/CDK1 complex formation necessary for G2/M transition, likely contributed to the observed cell cycle arrest and decreased tumorigenesis [43–49]. Correspondingly, the results of functional assays (MTS and BrdU) validated the decreased proliferative capacity of the Huh‐7 cells with the *KDM6A* overexpression. However, further comprehensive studies, including implementing multiple cell lines and in vivo models, are necessary to validate these findings.

The EMT process is a biological function that is ongoing in most cancers. During this process, epithelial cells with polarized, adherent, and tight junction characteristics lose their epithelial characteristics and acquire migratory, invasive, and spindle‐like characteristics that are characteristic of mesenchymal cells. MET is a cellular reprogramming mechanism, the reverse process of EMT [50–53]. c‐MYC, an oncogenic transcription factor, promotes the EMT process by regulating key markers, including downregulating E‐cadherin and upregulating N‐cadherin, while also interacting with multiple signaling pathways to enhance the invasive and metastatic potential of tumor cells [44, 46, 49]. The transcription factors SNAIL1, SNAIL2, and ZEB2 are major EMT inducers during hepatocarcinogenesis [54, 55]. By binding to the promoter of the *CDH1* (E‐cadherin) gene, *SNAIL1*, *SNAIL2*, and *ZEB2* suppress *CDH1* expression and increase *CDH2* (N‐cadherin) levels, reducing cell adhesion and promoting mesenchymal traits such as increased motility and invasion in cancer cells [54, 56, 57].

Increased expression of *KDM6A* in transduced Huh‐7 cells in our study was correlated with a significant change in their morphology. Indeed, transduced Huh‐7 cells displayed more round polygonal shapes, with smaller nuclei and nucleoli. These changes reflecting the MET process were confirmed by the significant upregulation of *CDH1* )an epithelial marker with adherens junctions that maintain cell–cell adhesion) and *ZO1* (an epithelial marker and tight junction protein involved in hepatocyte morphology, polarization, and epithelial function) [58]. Conversely, there was a downregulation of *c-MYC* and *CDH2*, a mesenchymal marker known to promote cellular migration and invasion. Additionally, the expression of *ZEB1* and *SNAIL2*, key transcription factors that drive EMT by repressing *CDH1* transcription and promoting the expression of *CDH2* and other mesenchymal markers, was also affected. Notably, this cadherin switch can induce *ZEB1* expression, thereby reinforcing the mesenchymal phenotype and EMT progression. Given that *ZO1* is involved in signal transmission in cell–cell junctions and cell growth, as well as in epithelial morphogenesis and differentiation [59, 60], it can be inferred that the overexpression of *ZO1* at the mRNA expression and protein levels in the *KDM6A* overexpressed cells has a considerable impact on cell junctions, proliferation, and differentiation. Our findings revealed the “cadherin switch” in transduced Huh‐7 cells, which is characterized by the upregulation of *CDH1* and the downregulation of *CDH2*. The association of this switch with tumor progression and metastasis has been demonstrated in many cancers [15, 61, 62]. Moreover, the scratch or wound healing assay showed a decline in the migration and scratch area after overexpression of *KDM6A*. All these results can imply a remarkable reversion in the EMT process.

HNF4*α* plays a critical role in the maturation and functionality of hepatocytes while its expression was reported to downregulate during hepatocarcinogenesis [63–65]. Beyond its role in hepatic cell differentiation and regulation of key markers such as ALB and AFP, *HNF4α* is also important in attenuating cancer‐associated characteristics and suppressing tumor progression. This tumor‐suppressive effect is mediated through the inhibition of hepatocyte proliferation via repression of promitogenic gene expression, as well as by suppressing the EMT. Moreover, the EMT to MET switch regulated by *HNF4α* contributes to the maintenance of epithelial cell polarity and regulation of tight junction proteins [64, 66–68]. Notably, recent work by Shokouhian et al. proposed that *HNF4α* may serve as a crucial nexus linking EMT and the Warburg effect, two key processes driving hepatocarcinogenesis, thereby coordinating metabolic reprogramming with cellular phenotype changes [64]. Additionally, their comprehensive analysis of hepatogenesis and hepatocarcinogenesis signaling pathways provides further insight into the complex regulatory network in which HNF4*α* is a key operator [69].

The *HNF4α* downregulation in liver cancer was also associated with EMT progression and poor prognosis [64, 68, 70, 71]. Studies exploring *HNF4α* induction demonstrated that this transcription factor inhibits liver cell proliferation [64, 72], while its inhibition promotes tumorigenesis in liver and colorectal cancers [58]. Previously, Shokouhian et al. showed that *HNF4α* links cancer metabolism with the EMT process in HCC cells, where its upregulation can partially restore the epithelial morphology of cells with normal metabolism [64]. Similarly, Miri‐Lavasani et al. demonstrated that activation of *HNF4α* by its natural ligand reduces the cancerous phenotype of HCC cells, decreasing EMT, migration, and proliferation [68].

In this study, we showed the HNF4*α* upregulation at mRNA and protein levels after *KDM6A* overexpression. The impact of such upregulation was noticed at the functional levels as demonstrated by the increased ALB and decreased AFP secretions in the *KDM6A* overexpression group.

*HNF4α* is also a critical regulator of key metabolic enzymes CYP3A4 and CYP2E1 [73–75], both essential members of the cytochrome P450 family involved in the metabolism of drugs, toxins, hormones, and endogenous compounds [73, 76–78]. Mutations or disruptions in HNF4A expression can alter CYP3A4 and CYP2E1 levels, impacting drug pharmacokinetics and hepatotoxicity susceptibility (96, 98‐101). Additionally, ALB is involved in drug distribution and activity by modulating free drug concentrations (103, 104), and its expression is indirectly influenced by *HNF4α* through transcriptional regulation. Both *ALB* and *AFP* genes contain promoter and enhancer regions targeted by *HNF4α*, which acts in concert with other liver‐specific transcription factors to regulate their expression.

In our study, overexpression of *KDM6A* led to a significant upregulation of *HNF4α* at mRNA and protein levels, alongside increased mRNA expression of *ALB*, *CYP3A4*, and *CYP2E1*. Functionally, this was reflected by elevated ALB secretion and decreased AFP secretion in the *KDM6A*‐overexpressing cells, indicating enhanced hepatocyte maturation. The increased expression of *CYP3A4* and *CYP2E1* further suggests improved metabolic activity and functional maturation, which contribute to reduced proliferation and migratory behavior of the cancer cells.

Moreover, modulating *KDM6A* activity, either through down‐ or upregulation, can significantly disrupt the balance of H3K27me3 and H3K27ac, leading to widespread changes in developmental, differentiation, and cell‐cycle pathways. This suggests that chromatin remodeling is not just confined to the intended locus, and for that reason, it is important to also assess related regulators such as KDM6B/UTX2 and PRC2 components, together with broader epigenetic analyses, which is warranted [7, 8, 41, 79–81]. Moreover, since KDM6A also exerts noncatalytic functions by serving as a scaffold within transcriptional complexes, its perturbation may interfere with coregulatory networks independently of its demethylase activity [7, 13, 82–84].

Limitations of this study include reliance on overexpression models restricted to in vitro Huh‐7 cells. Future work should incorporate loss‐of‐function experiments, validation of results in diverse hepatoma cell lines, and in vivo models to confirm the universality and therapeutic potential of *KDM6A* modulation in the future. Additionally, therapeutic targeting strategies such as siRNA or small molecule inhibitors of *KDM6A* or its effectors could provide mechanistic insight and translational relevance.

While our findings support a tumor‐suppressive role for *KDM6A* in HCC, it is essential to consider potential challenges associated with therapeutic modulation of this epigenetic regulator in vivo. Modulating *KDM6A* activity may lead to unintended off‐target effects due to its broad influence on chromatin remodeling and gene expression across multiple pathways. Given *KDM6A*’s involvement in biological processes such as development, differentiation, and hematopoiesis, systemic consequences and tissue‐specific responses need to be carefully evaluated [16, 85]. Furthermore, as *KDM6A* also functions via noncatalytic mechanisms within transcriptional complexes, its perturbation may affect coregulatory networks beyond alterations in H3K27 methylation status [19]. Therefore, comprehensive in vivo studies, as well as assessment of off‐target and systemic effects, are necessary before considering *KDM6A*‐targeted therapies for clinical translation.

In conclusion, our findings demonstrate that *KDM6A* overexpression modulates malignant phenotypes in HCC cells by inhibiting proliferation, disrupting EMT, and promoting hepatic differentiation through the regulation/modulation of key genes including *c-MYC*, *CCNB1*, *HNF4α*, and downstream targets. These data support the role of *KDM6A* as a tumor suppressor and highlight its potential as a target for differentiation‐based therapies in HCC.

**Nomenclature**
AFP: alpha‐fetoprotein
ALB: albumin
*CCNB1*
: cyclin B1
*CDH1*
: Cadherin 1
*CDH2*
: Cadherin 2
EMT: epithelial–mesenchymal transition
PPI: protein–protein interaction
HCC: hepatocellular carcinoma
*HNF4α*
: hepatocyte nuclear factor 4 alpha
*KDM6A*
: lysine demethylase 6A
*PROM1*
: Prominin 1
*C-MYC*
: cellular myelocytomatosis oncogene
*ZEB1*
: zinc‐finger E‐box‐binding homeobox 1
ZO1: zonula occludens protein 1
CTRL: control group
DMEM: Dulbecco’s Modified Eagle Medium
PCR: polymerase chain reaction
BrdU: 5‐bromodeoxyuridine
qPCR: quantitative polymerase chain reaction
ELISA: enzyme‐linked immunosorbent assay
BCA: bicinchoninic acid assay
SDS‐PAGE: sodium dodecyl sulfate polyacrylamide gel electrophoresis
CYPs: cytochromes P450

## Ethics Statement

This study was confirmed in Royan Institute Ethics Committee by the number IR.ACECR.ROYAN.REC.1400.154b.

## Disclosure

Abbas Piryaei, Mustapha Najimi, and Massoud Vosough approved the manuscript.

## Conflicts of Interest

The authors declare no conflicts of interest.

## Author Contributions

Mahdieh Hashemi performed the experiments, data collection, and data analysis, drafted the manuscript, and prepared the figures. Mahdi Hesaraki and Abbas Piryaei reviewed and edited the draft. Bahare Shokouhian edited the manuscript and PPI network analysis. Roya Ramezankhani contributed to the conceptualization and performed the vector construction. Seyyed Mohammad Yaghoubi prepared the graphical abstract and reviewed and edited the manuscript. Elham Rismani critically reviewed and edited the manuscript. Abbas Piryaei, Mustapha Najimi, and Massoud Vosough developed the concept and contributed to the data analysis and critical reviewing. Massoud Vosough contributed to the financial support of the project.

## Funding

The study was funded by Royan Institute (10.13039/501100004920, 400000032) and Bahar Tashkhis Teb (BT14000211).

## Supporting information


**Supporting Information** Additional supporting information can be found online in the Supporting Information section. Additional supporting data are provided in the Supporting Information (Supplementary_R.pdf). Table S1 lists the primers used in this study. Figure S1 presents the validation of KDM6A overexpression, gene expression analyses, cell viability assays, and Western blot results supporting the main findings. Figure S2 shows the morphological changes of cells following KDM6A overexpression and their reversion after subsequent passages. Detailed descriptions of each table and figure are provided in the Supplementary_R.pdf.

## Data Availability

Data are available on request from corresponding authors.
