# Supplementary material for: Overexpression of KDM6A in Hepatoma Cells Induces Hepatocytic Differentiation and Attenuates Proliferation Rate, Colony Formation, and Migration Capacities
Source: Biomed Res Int. 2025 Nov 15;2025:5551687. doi: 10.1155/bmri/5551687 (PMC12619137; doi:10.1155/bmri/5551687)
Supplement: Supplementary file 1 — Supporting Information Additional supporting information can be found online in the Supporting Information section. Additional supporting data are provided in the Supporting Information (Supplementary_R.pdf). Table S1 lists the primers used in this study. Figure S1 presents the validation of KDM6A overexpression, gene expression analyses, cell viability assays, and Western blot results supporting the main findings. Figure S2 shows the morphological changes of cells following KDM6A overexpression and their reversion after subsequent passages. Detailed descriptions of each table and figure are provided in the Supplementary_R.pdf. [file BMRI-2025-5551687-s001.pdf]

Table S1. List of the primers used in the study

Gene	Primer: Forward (F) and Reverse (R)
KDM6A	F: 5' GGACATGCTGTGTCACATCCT 3'
	R: 5' CTCCTGTTGGTCTCATTTGGTG 3'
GAPDH	F: 5' CTCATTTCCTGGTATGACAACGA 3'
	R: 5' CTTCCTCTTGTGCTCTTGCT 3'
HISTON H1b1	F: 5' CGACGTGGAGAAGAATAACAG 3'
	R: 5' TTTAAAGGAGCCAGAAGCAC 3'
CDH1	F: 5'AATCACATCCTACACTGCCC 3’
	R: 5' GCAACTGGAGAACCATTGTC 3'
CDH2	F: 5' AGCCAACCTTAACTGAGGAGT 3’
	R: 5' GGCAAGTTGATTGGAGGGATG 3'
PROM1	F: 5' AGTCGGAAACTGGCAGATAGC 3'
	R: 5' GGTAGTGTTGTACTGGGCCAAT 3'
C-MYC	F : 5' GTCAAGAGGCGAACACACAAC 3'
	R: 5' TTGGACGGACAGGATGTATGC 3'
ZEB1	F:5' TTCACAATTACTCACCTGTCCA 3'
	R: 5' TGCGTCACATGTCTTTGATCTC 3'
SNAIL1	F: 5' CAATCGGAAGCCTAACTACAGC 3'
	R: 5' GACAGAGTCCCAGATGAGCA 3'
SNAIL2	F- 5' CGAACTGGACACACATACAGTG 3'
	R- 5' CTGAGGATCTCTGGTTGTGGT 3'
HNF4A	F 5' CGA TGA CAA TGA GTA TGC CT 3'
	R: 5' GTC GTT GAT GTA GTC CTC CA 3'
ZO1	F: 5' CTGGTGAAATCCCGGAAAAATGA 3'
	R: 5' TTGCTGCCAAACTATCTTGTGA 3'

ALBUMIN	F: 5' CTGCTTGAATGTGCTGATGACAG 3'<br>R: 5'GGCATAGCATTCATGAGGATCTG 3'
CCNB1	F: 5' GCT GGG TGT AGG TCC TTG 3'<br>R: 5' CCT GCC ATG TTG ATC TTC G 3'

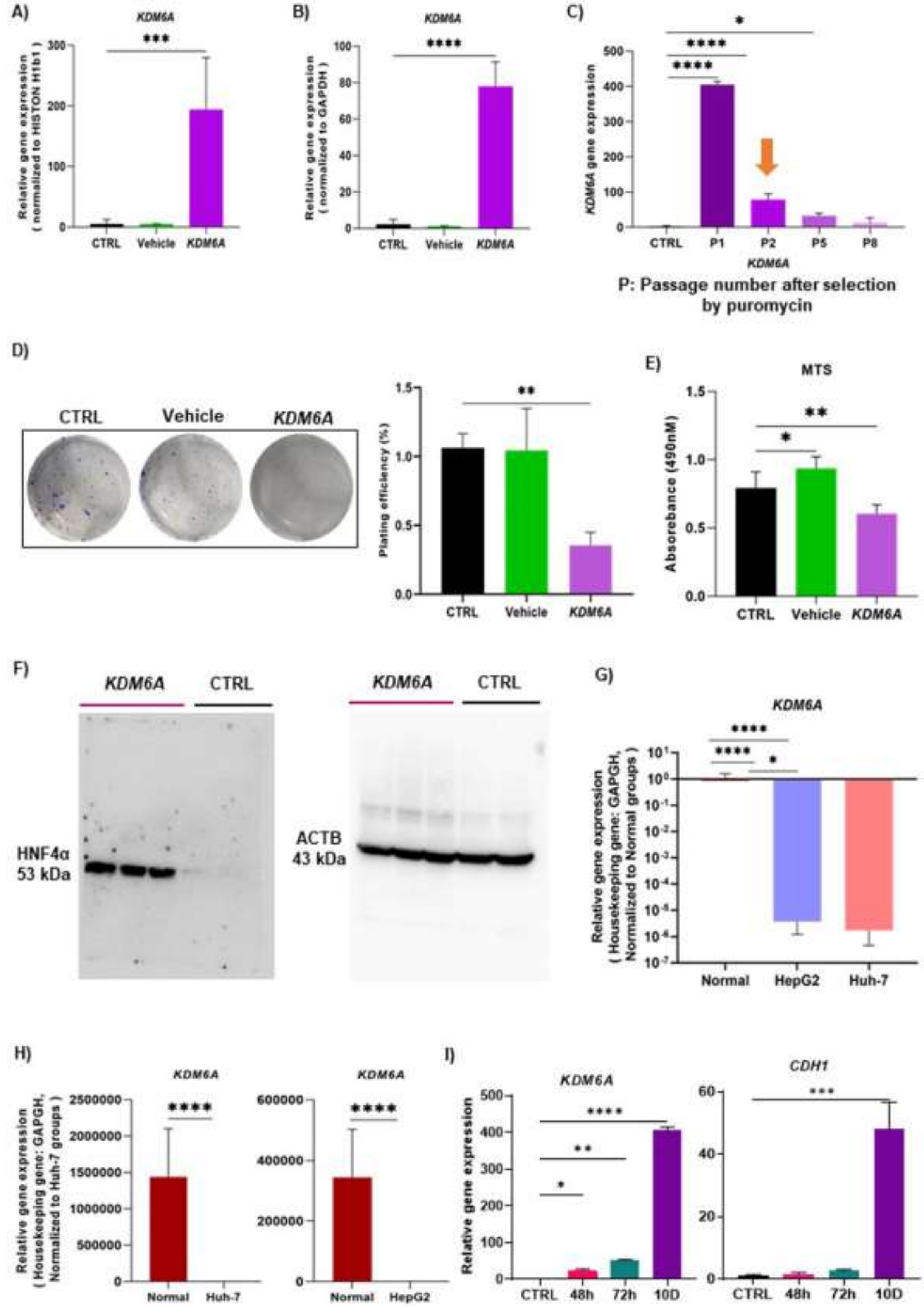

A)
KDM6A
***
Relative gene expression
(normalized to HISTON H1b1)
300
200
100
0
CTRL
Vehicle
KDM6A
B)
KDM6A
****
Relative gene expression
(normalized to GAPDH)
100
80
60
40
20
0
CTRL
Vehicle
KDM6A
C)
*
****
****
KDM6A gene expression
500
400
300
200
100
0
CTRL
P1
P2
P5
P8
KDM6A
P: Passage number after selection
by puromycin
D)
CTRL
Vehicle
KDM6A
**
Plating efficiency (%)
1.5
1.0
0.5
0.0
CTRL
Vehicle
KDM6A
E)
MTS
**
*
Absorebance (490nM)
1.5
1.0
0.5
0.0
CTRL
Vehicle
KDM6A
F)
KDM6A
CTRL
HNF4α
53 kDa
KDM6A
CTRL
ACTB
43 kDa
G)
KDM6A
****
****
*
Relative gene expression
(Housekeeping gene: GAPGH,
Normalized to Normal groups)
Normal
HepG2
Huh-7
H)
KDM6A
****
Relative gene expression
(Housekeeping gene: GAPGH,
Normalized to Huh-7 groups)
2500000
2000000
1500000
1000000
500000
0
Normal
Huh-7
KDM6A
****
600000
400000
200000
0
Normal
HepG2
I)
KDM6A
****
**
*
Relative gene expression
500
400
300
200
100
0
CTRL
48h
72h
10D
CDH1
***
60
40
20
0
CTRL
48h
72h
10D

Supplementary Figure 1: A, and B) Measuring the expression of the *KDM6A* in the groups treated with the vector carrying the *KDM6A* gene, the empty vector group and the control group indicated a significant increase in the *KDM6A* gene compared to the empty vector (vehicle) and CTRL groups. *KDM6A* expression in the empty vector groups was not significant in comparison to the CTRL group. The *KDM6A* expression was evaluated and normalized to *GAPDH* and *HISTON H1b1* housekeeping genes. C) After the cells were selected with puromycin antibiotic at different times, *KDM6A* expression was checked in different passages after transduction, and approximately cells P1 and P2 were chosen to perform the tests. D) *KDM6A* induction resulted in impaired cell growth and a decline in plating efficiency percentage and colony-forming ability of the Huh-7 cell compared to the CTRL and vehicle groups. E) MTS assays: decrease detected the cell viability compared with the vehicle and CTRL group. F) Full scan image hole blot Western blot assay HNF4α: 54kDa and ACTB: 43kDa. G) Quantitative RT-PCR analysis revealed that *KDM6A* expression was significantly downregulated in HCC cell lines (HepG2 and Huh-7) compared to normal liver cells, based on normalization to *GAPDH* as the housekeeping gene. H) Quantitative RT-PCR results confirmed markedly lower expression levels of *KDM6A* in the Huh-7 and HepG2 HCC lines relative to normal liver cells, with statistical significance indicated by ****P<0.0001. I) Induction of *KDM6A* in cells at various timepoints post-transduction (48h, 72h, and 10 days) demonstrated a significant increase in *KDM6A* expression at day 10, which was paralleled by upregulation of *CDH1* gene expression, compared to control and earlier timepoints. Data are presented as the One-way-ANOVA analysis, the mean ± SD for three biological independent repeated experiments. *****P-value*<0.0001, ****P-value*<0.001 , ***P-value*<0.01, **P-value*<0.05.

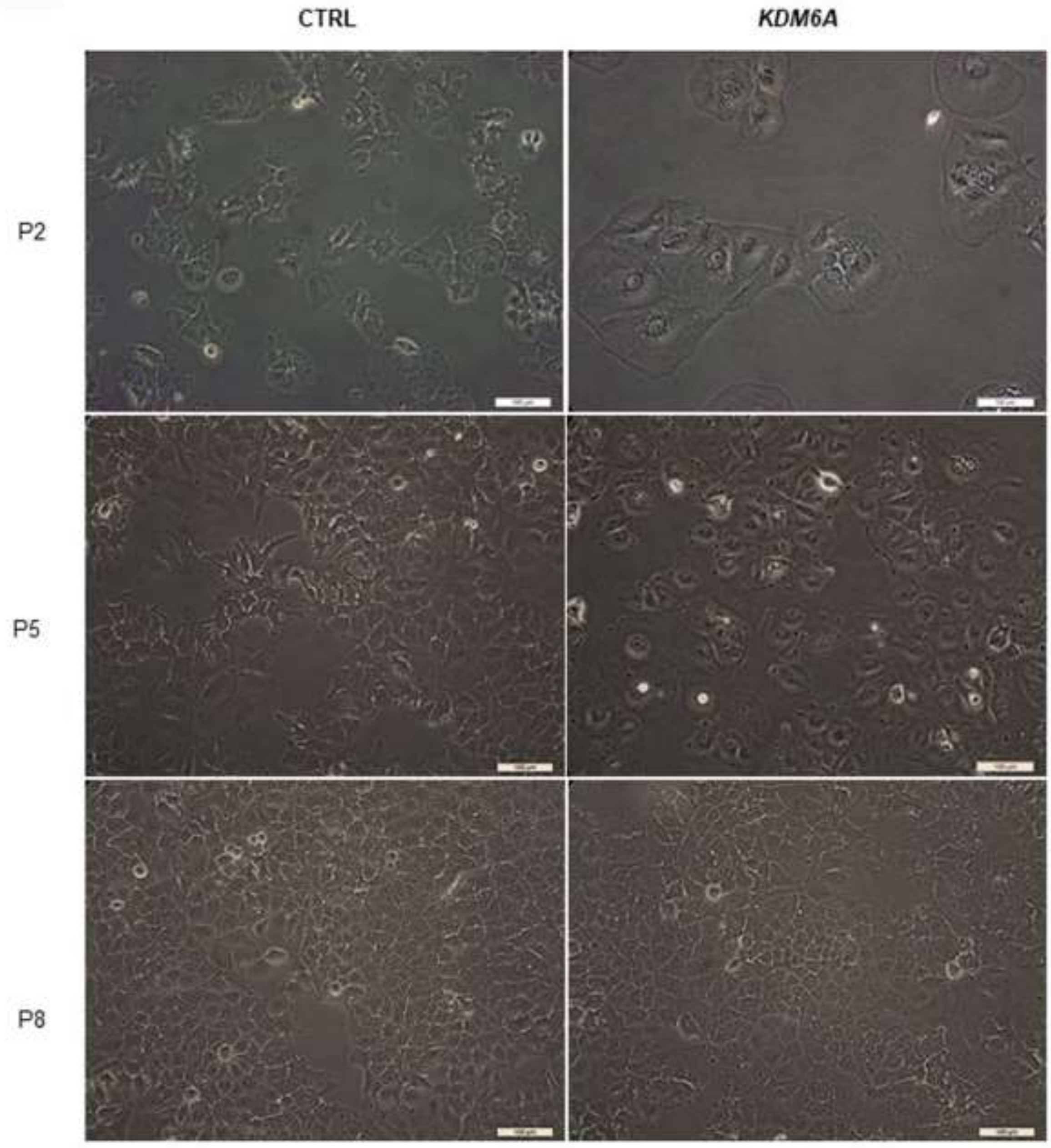

Supplementary Figure 2: After transducing and overexpression of *KDM6A*, the morphology of the cells changed to more epithelial forms. The *KDM6A* overexpressing cells showed round polygonal shapes, the nucleus and nucleoli became smaller, and the cells' cytoplasm was higher than the control cells. Also, the transduced cells returned to their initiate morphology with

increased cell passage number after selection with puromycin. P: Passage number after selection by puromycin.
